# Supplementary material for: Utilization of Bioflocculants from Flaxseed Gum and Fenugreek Gum for the Removal of Arsenicals from Water
Source: Materials (Basel). 2022 Dec 6;15(23):8691. doi: 10.3390/ma15238691 (PMC9740097; doi:10.3390/ma15238691)
Supplement: Supplementary file 1 [file materials-15-08691-s001.zip › materials-2002676-supplementary.pdf]

SUPPLEMENTARY MATERIALS

Utilization of Bioflocculants from Flaxseed Gum and Fenugreek Gum for the Removal of Arsenicals from Water

Deysi J. Venegas-García and Lee D. Wilson *

Department of Chemistry, University of Saskatchewan, 110 Science Place, Thorvaldson Building (Room 165), Saskatoon, SK S7N 5C9, Canada
* Correspondence: lee.wilson@usask.ca

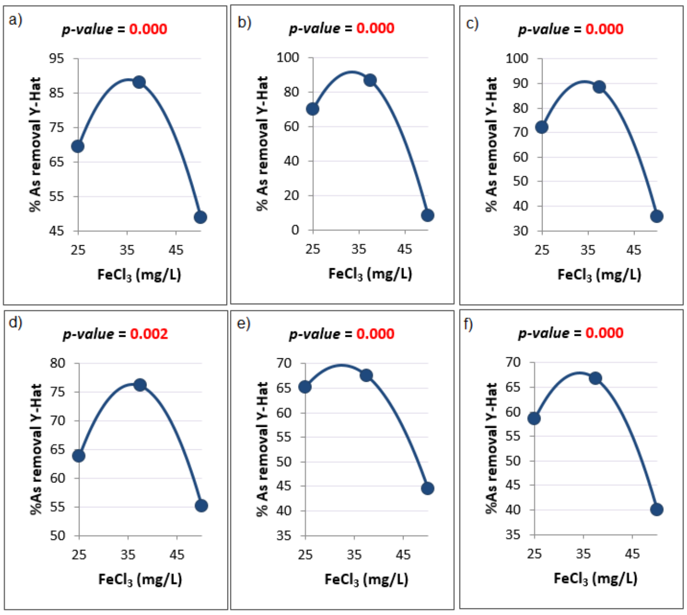

Figure S1. Coagulant (mg/L $FeCl_3$) effect for **S1** (Roxarsone) arsenic removal with FSG (a), FGG (b) and XG (c), and **S2** arsenic removal with FSG (d), FGG (e) and XG (f). Probability values (p values) less than 0.05 are considered to be statistically significant.

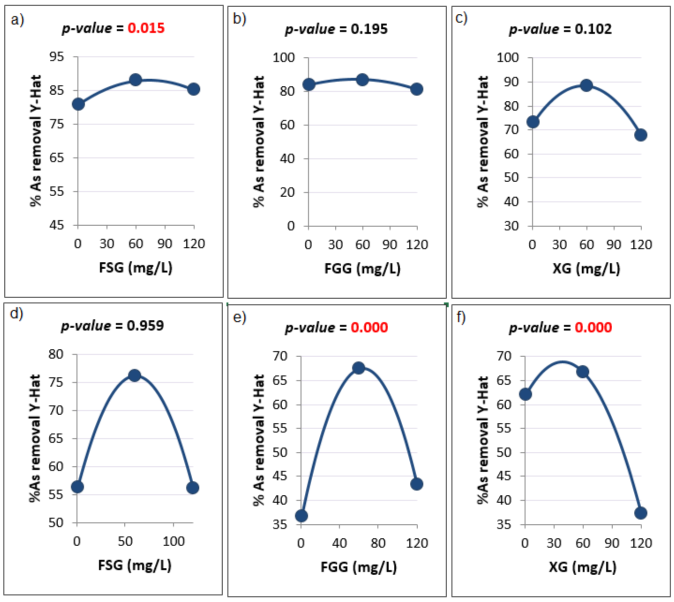

Figure S2. Flocculant dosage (mg/L) effect for **S1** (Roxarsone) arsenic removal with FSG (a), FGG (b) and XG (c), and **S2** arsenic removal with FSG (d), FGG (e) and XG (f). Probability values (p values) less than 0.05 are considered to be statistically significant.

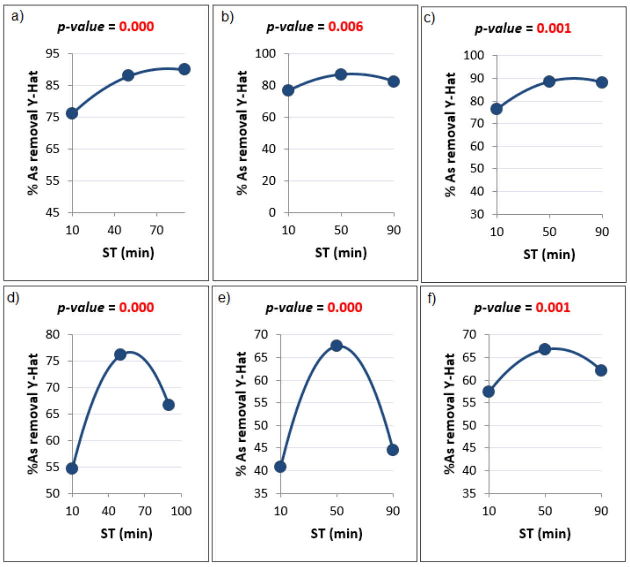

Figure S3. Settling time (mg/L) effect for **S1**(Roxarsone) arsenic removal with FSG (a), FGG (b) and XG (c), and **S2** arsenic removal with FSG (d), FGG (e) and XG (f). Probability values (p values) less than 0.05 are considered to be statistically significant.

Table S1. ANOVA table for arsenic removal (%) S1-FSG at 295 K.

SOURCE	SEQ SS	ADJ SS	DF	ADJ MS	F	P	R^2	0.979
**MAIN**	2,509.5		3				Adj R^2	0.9695
**COAGULANT (A)**	1,681.0	1,681.0	1	1,681.0	166.642	**0.000**	Std Error	3.1761
**FLOCCULANT (B)**	72.25	72.25	1	72.25	7.1623	**0.015**	F	103.523
**ST (C)**	756.25	756.25	1	756.25	74.969	**0.000**	Sig F	0.0
**2 - WAY**	603.375		3				FLOF	63.621
**AB**	435.125	435.125	1	435.125	43.135	**0.000**	Sig FLOF	0.0
**AC**	153.125	153.125	1	153.125	15.18	**0.001**		
**BC**	15.125	15.125	1	15.125	1.4994	0.235		
**QUADRATIC**	6,285.68		3					
**AA**	5,951.34	6,183.72	1	6,183.72	613.008	**0.000**		
**BB**	154.31	180.029	1	180.029	17.847	**0.000**		
**CC**	180.029	180.029	1	180.029	17.847	**0.000**		
**REGRESSION**	9,398.55	9,398.55	9	1,044.28				
**ERROR**	201.75	201.75	20	10.088				
**ERROR PURE**	16.5	16.5	17	0.9706				
**ERROR LOF**	185.25	185.25	3	61.75				
**TOTAL**	9,600.3		29					

Seq SS, sequential sum of squares; Adj SS, adjusted sum of squares; d.f., degree of freedom; Adj MS, adjusted mean square; *F*, *F* statistic for the term; *P*, *P* value for the term (95% confidence).

Table S2. ANOVA table for arsenic removal (%) S1-FGG at 295 K.

SOURCE	SEQ SS	ADJ SS	DF	ADJ MS	F	P	R^2	**0.9907**
**MAIN**	15,485.6		3				Adj R^2	0.9865
**COAGULANT (A)**	15,314.1	15,314.1	1	15,314.1	998.065	**0.000**	Std Error	3.9171
**FLOCCULANT (B)**	27.563	27.563	1	27.563	1.7963	0.195	F	235.772
**ST (C)**	144	144	1	144	9.3849	**0.006**	Sig F	0
**2 - WAY**	37.75		3				F_{LOF}	90.942
**AB**	12.5	12.5	1	12.5	0.8147	0.377	Sig F_{LOF}	0.0
**AC**	15.125	15.125	1	15.125	0.9857	0.333		
**BC**	10.125	10.125	1	10.125	0.6599	0.426		
**QUADRATIC**	17,035.2		3					
**AA**	16,512.7	16,925.7	1	16,925.7	1,103.1	**0.000**		
**BB**	107.16	141.346	1	141.346	9.212	**0.007**		
**CC**	415.385	415.385	1	415.385	27.072	**0.000**		
**REGRESSION**	32,558.6	32,558.6	9	3,617.62				
**ERROR**	306.875	306.875	20	15.344				
**ERROR PURE**	18	18	17	1.0588				
**ERROR LOF**	288.875	288.875	3	96.292				
**TOTAL**	32,865.5		29					

Seq SS, sequential sum of squares; Adj SS, adjusted sum of squares; d.f., degree of freedom; Adj MS, adjusted mean square; *F*, *F* statistic for the term; *P*, *P* value for the term (95% confidence).

Table S3. ANOVA table for arsenic removal (%) S1-XG at 295 K.

SOURCE	SEQ SS	ADJ SS	DF	ADJ MS	F	P	R^2	**0.9583**
**MAIN**	6,008.63		3				Adj R^2	0.9396
**COAGULANT (A)**	5,329.0	5,329.0	1	5,329.0	135.232	**0.000**	Std Error	6.2774
**FLOCCULANT (B)**	115.563	115.563	1	115.563	2.9326	0.102	F	51.108
**ST (C)**	564.063	564.063	1	564.063	14.314	**0.001**	Sig F	0.0
**2 - WAY**	1,451.12		3				F_{LOF}	257.042
**AB**	1,352.0	1,352.0	1	1,352.0	34.309	**0.000**	Sig F_{LOF}	0.0
**AC**	8.0	8.0	1	8.0	0.203	0.657		
**BC**	91.125	91.125	1	91.125	2.3125	0.144		
**QUADRATIC**	10,666.0		3					
**AA**	8,034.69	8,821.41	1	8,821.41	223.858	**0.000**		
**BB**	2,325.27	2,442.72	1	2,442.72	61.988	**0.000**		
**CC**	306.029	306.029	1	306.029	7.766	**0.011**		
**REGRESSION**	18,125.7	18,125.7	9	2,013.97				
**ERROR**	788.125	788.125	20	39.406				
**ERROR PURE**	17	17	17	1				
**ERROR LOF**	771.125	771.125	3	257.042				
**TOTAL**	18,913.9		29					

Seq SS, sequential sum of squares; Adj SS, adjusted sum of squares; d.f., degree of freedom; Adj MS, adjusted mean square; *F*, *F* statistic for the term; *P*, *P* value for the term (95% confidence).

Table S4. ANOVA table for arsenic removal (%) S2-FSG at 295 K.

SOURCE	SEQ SS	ADJ SS	DF	ADJ MS	F	P	R^2	**0.9407**
**MAIN**	873.625		3				**Adj R^2**	0.914
**COAGULANT (A)**	297.562	297.562	1	297.562	12.643	**0.002**	**Std Error**	4.8513
**FLOCCULANT (B)**	0.0625	0.0625	1	0.0625	0.0027	0.959	**F**	35.226
**ST (C)**	576.0	576.0	1	576.0	24.474	**0.000**	**Sig F**	0.0
**2 - WAY**	681.25		3				**F_{LOF}**	240.55
**AB**	392.0	392.0	1	392.0	16.656	**0.001**	**Sig F_{LOF}**	0.0
**AC**	36.125	36.125	1	36.125	1.5349	0.230		
**BC**	253.125	253.125	1	253.125	10.755	**0.004**		
**QUADRATIC**	5,906.72		3					
**AA**	1,499.15	2,061.55	1	2,061.55	87.594	**0.000**		
**BB**	2,614.29	2,941.55	1	2,941.55	124.984	**0.000**		
**CC**	1,793.28	1,793.28	1	1,793.28	76.195	**0.000**		
**REGRESSION**	7,461.59	7,461.59	9	829.066				
**ERROR**	470.708	470.708	20	23.535				
**ERROR PURE**	10.833	10.833	17	0.6373				
**ERROR LOF**	459.875	459.875	3	153.292				
**TOTAL**	7,932.3		29					

Seq SS, sequential sum of squares; Adj SS, adjusted sum of squares; d.f., degree of freedom; Adj MS, adjusted mean square; *F*, *F* statistic for the term; *P*, *P* value for the term (95% confidence).

Table S5. ANOVA table for arsenic removal (%) S2-FGG at 295 K.

SOURCE	SEQ SS	ADJ SS	DF	ADJ MS	F	P
**MAIN**	1,923.13		3			
**COAGULANT (A)**	1,701.56	1,701.56	1	1,701.56	598.352	**0.000**
**FLOCCULANT (B)**	169.0	169.0	1	169.0	59.429	**0.000**
**ST (C)**	52.562	52.562	1	52.562	18.484	**0.000**
**2 - WAY**	176.25		3			
**AB**	171.125	171.125	1	171.125	60.176	**0.000**
**AC**	2.0	2.0	1	2.0	0.7033	0.412
**BC**	3.125	3.125	1	3.125	1.0989	0.307
**QUADRATIC**	10,021.9		3			
**AA**	589.301	1,177.04	1	1,177.04	413.904	**0.000**
**BB**	4,863.27	5,584.62	1	5,584.62	1,963.82	**0.000**
**CC**	4,569.35	4,569.35	1	4,569.35	1,606.8	**0.000**
**REGRESSION**	12,121.3	12,121.3	9	1,346.81		
**ERROR**	56.875	56.875	20	2.8438		
**ERROR PURE**	7.5	7.5	17	0.4412		
**ERROR LOF**	49.375	49.375	3	16.458		
**TOTAL**	12,178.2		29			

R^2	**0.9953**
**Adj R^2**	0.9932
**Std Error**	1.6863
**F**	473.604
**Sig F**	0
**F_{LOF}**	37.306
**Sig F_{LOF}**	0.0

Seq SS, sequential sum of squares; Adj SS, adjusted sum of squares; d.f., degree of freedom; Adj MS, adjusted mean square; *F*, *F* statistic for the term; *P*, *P* value for the term (95% confidence).

Table S6. ANOVA table for arsenic removal (%) S2-XG at 295 K.

SOURCE	SEQ SS	ADJ SS	DF	ADJ MS	F	P
**MAIN**	3,886.38		3			
**COAGULANT (A)**	1,350.56	1,350.56	1	1,350.56	234.456	**0.000**
**FLOCCULANT (B)**	2,450.25	2,450.25	1	2,450.25	425.36	**0.000**
**ST (C)**	85.563	85.563	1	85.563	14.854	**0.001**
**2 - WAY**	188.625		3			
**AB**	128.0	128.0	1	128.0	22.221	**0.000**
**AC**	0.125	0.125	1	0.125	0.0217	0.884
**BC**	60.5	60.5	1	60.5	10.503	**0.004**
**QUADRATIC**	4,208.76		3			
**AA**	1,837.53	2,234.7	1	2,234.7	387.94	**0.000**
**BB**	2,020.07	2,139.39	1	2,139.39	371.395	**0.000**

R^2	**0.9863**
**Adj R^2**	0.9801
**Std Error**	2.4001
**F**	159.783
**Sig F**	0.0
**F_{LOF}**	19.605
**Sig F_{LOF}**	0.0

**CC**	351.157	351.157	1	351.157	60.96	**0.000**
**REGRESSION**	8,283.76	8,283.76	9	920.418		
**ERROR**	115.208	115.208	20	5.7604		
**ERROR PURE**	25.833	25.833	17	1.5196		
**ERROR LOF**	89.375	89.375	3	29.792		
**TOTAL**	8,398.97		29			

Seq SS, sequential sum of squares; Adj SS, adjusted sum of squares; d.f., degree of freedom; Adj MS, adjusted mean square; *F*, *F* statistic for the term; *P*, *P* value for the term (95% confidence).

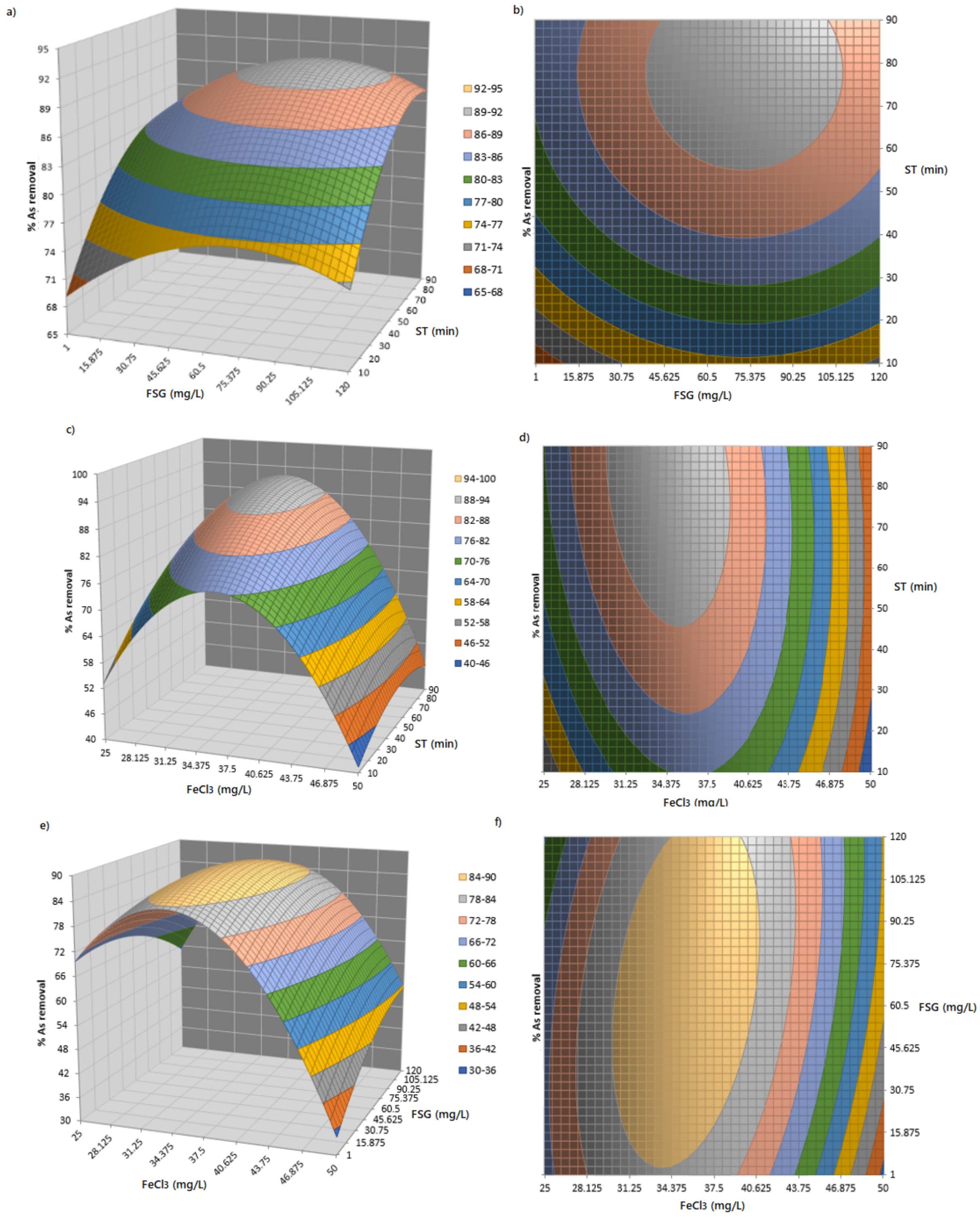

Figure S4. 3D Box-Behnken response surface (a, c, e) and 2D contour plots (b, d, f) of arsenic removal (S1) with FSG as a function of flocculant and settling time (a, b), coagulant and settling time (c, d), and coagulant and flocculant (e, f).

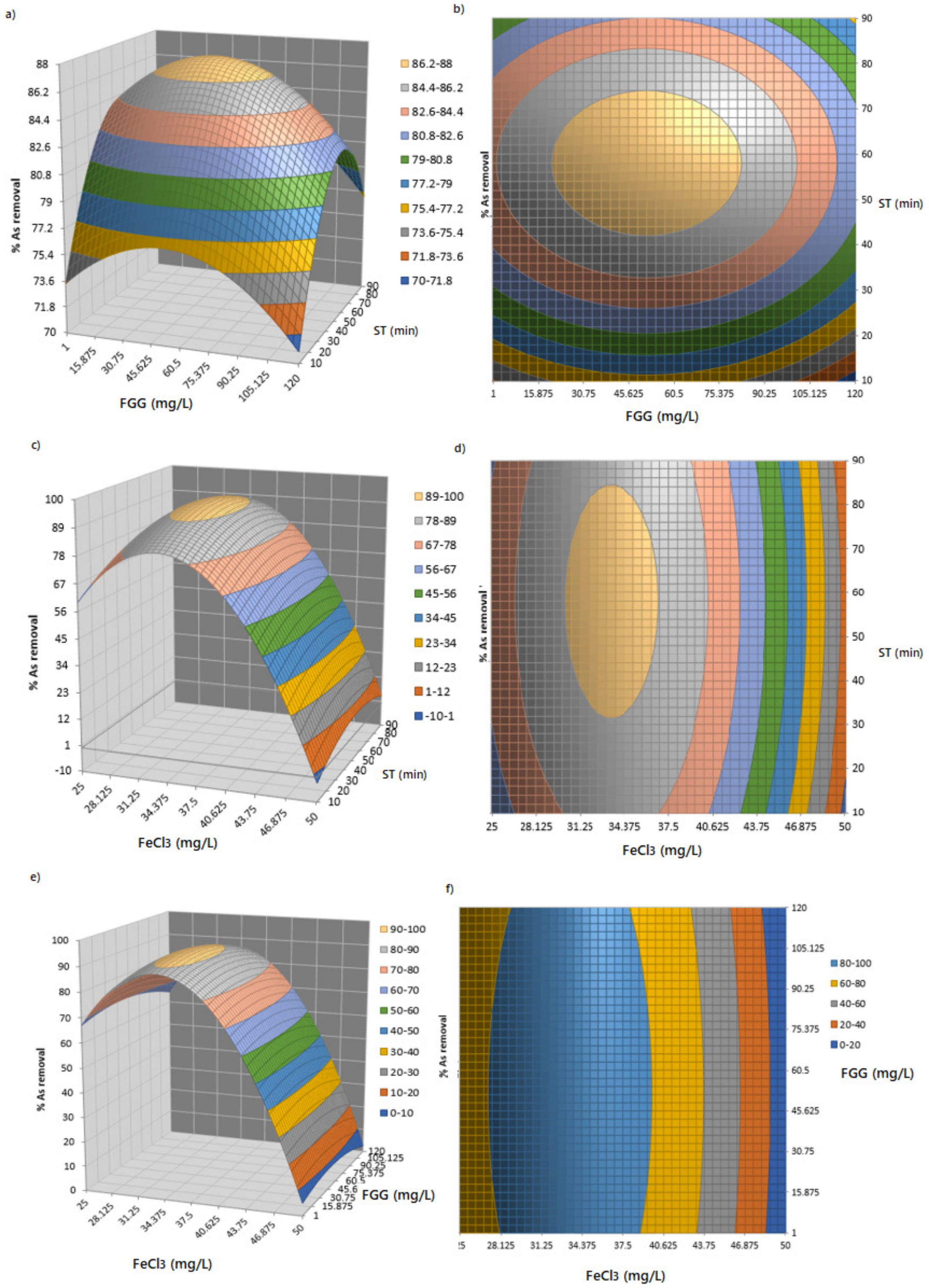

Figure. S5. 3D Box-Behnken response surface (a, c, e) and 2D contour plots (b, d, f) of arsenic removal (S1) with FGG as a function of flocculant and settling time (a, b), coagulant and settling time (c, d), and coagulant and flocculant (e, f).

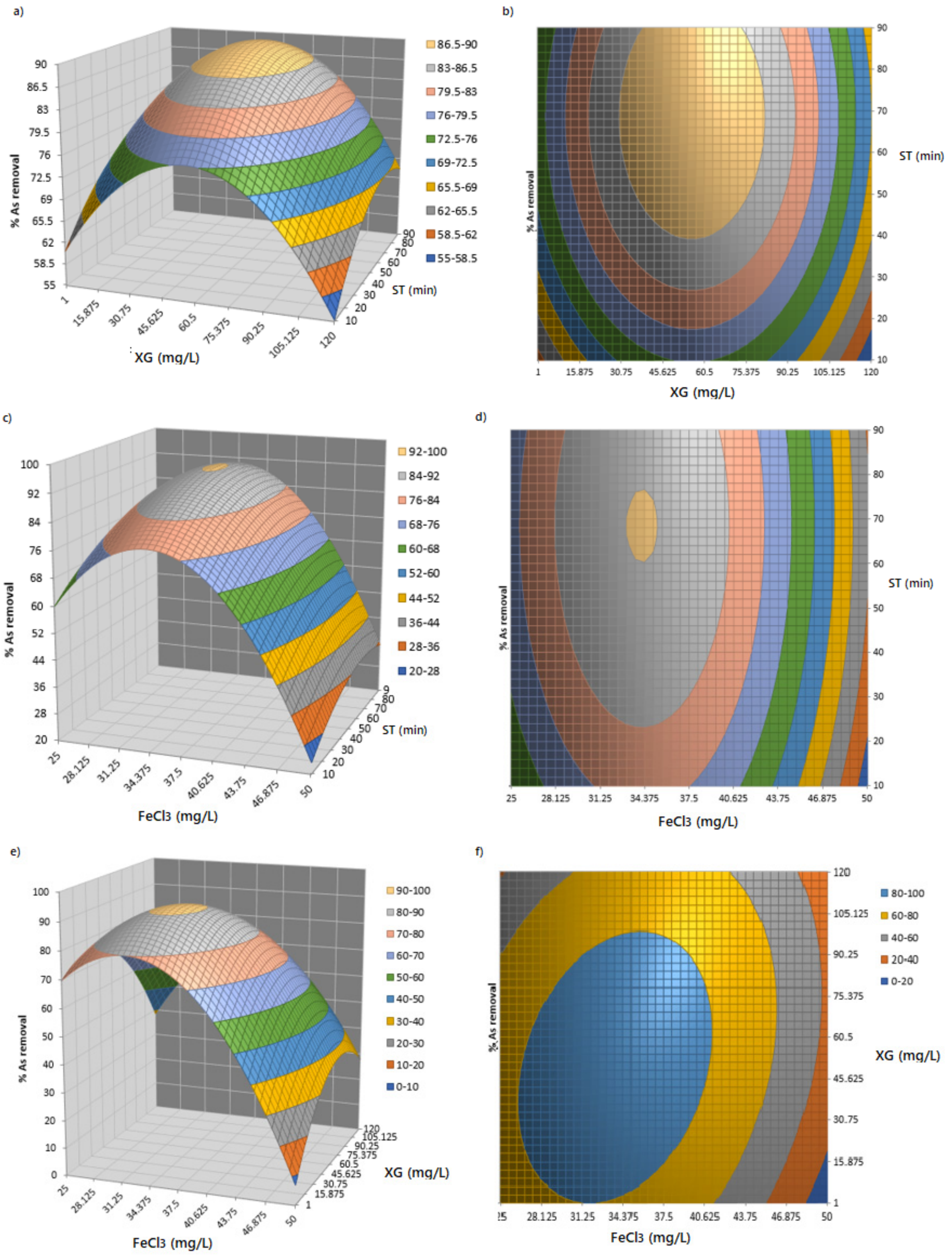

Figure S6. 3D Box-Behnken response surface (a, c, e) and 2D contour plots (b, d, f) of arsenic removal (S1) with XG as a function of flocculant and settling time (a, b), coagulant and settling time (c, d), and coagulant and flocculant (e, f).

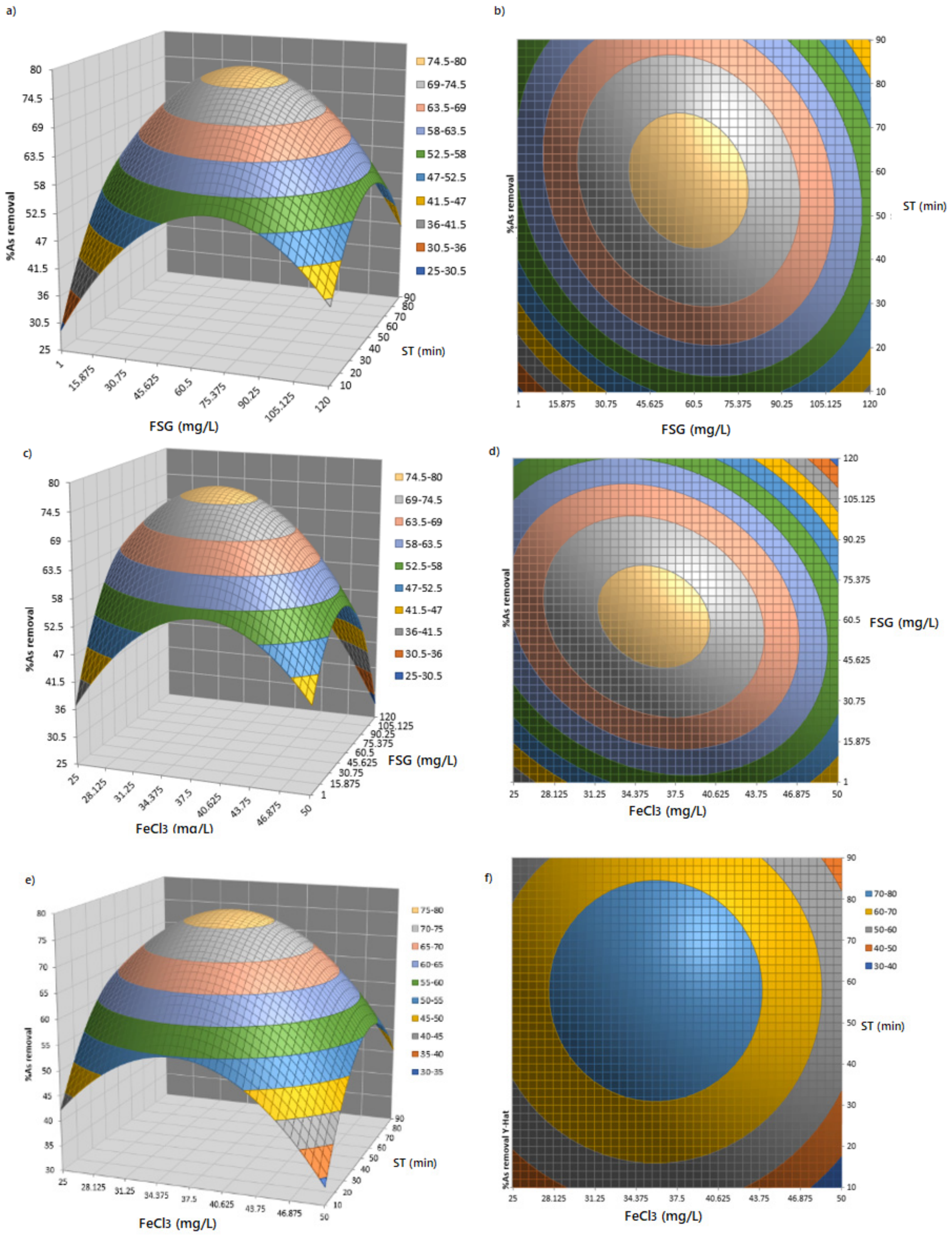

Figure S7. 3D Box-Behnken response surface (a, c, e) and 2D contour plots (b, d, f) of arsenic removal (S2) with FSG as a function of flocculant and settling time (a, b), coagulant and settling time (c, d), and coagulant and flocculant (e, f).

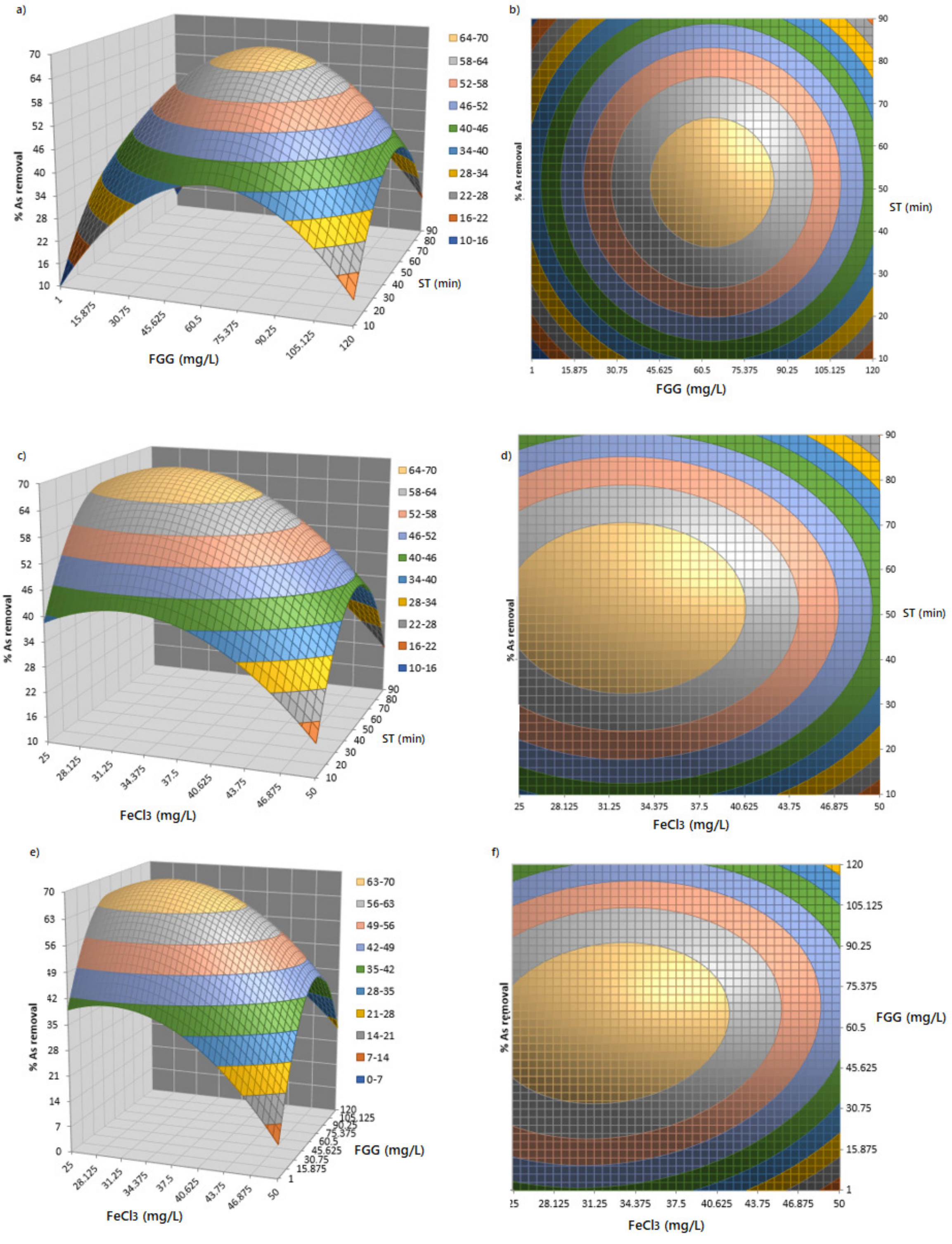

Figure S8. 3D Box-Behnken response surface (a, c, e) and 2D contour plots (b, d, f) of arsenic removal (S2) with FGG as a function of flocculant and settling time (a, b), coagulant and settling time (c, d), and coagulant and flocculant (e, f).

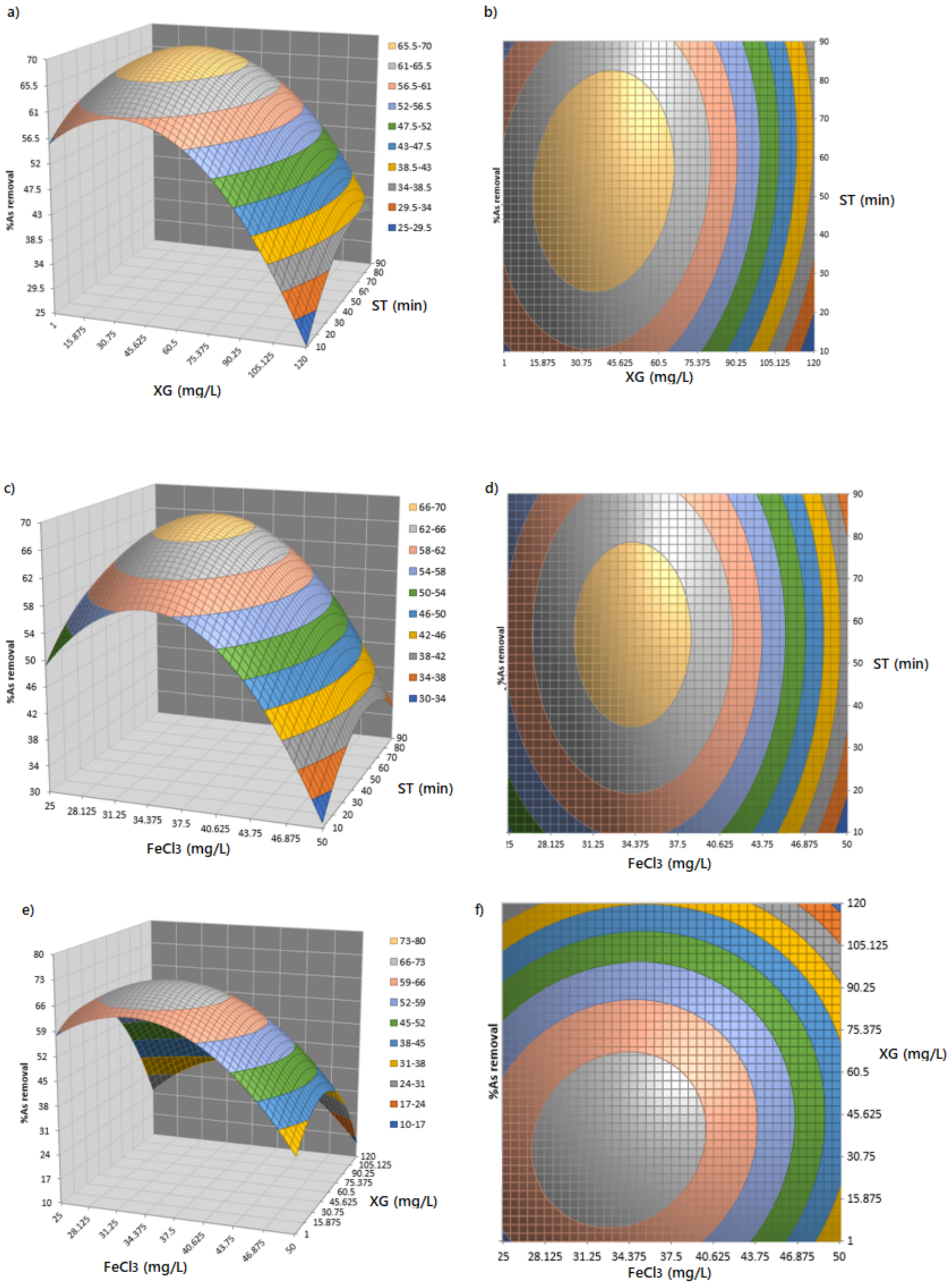

Figure S9. 3D Box-Behnken response surface (a, c, e) and 2D contour plots (b, d, f) of arsenic removal (S2) with XG as a function of flocculant and settling time (a, b), coagulant and settling time (c, d), and coagulant and flocculant (e, f).

Table S7. Box-Behnken experimental design matrix of three variables along with the experimental and calculated removal efficiency (S1; Roxarsone) at 295 K.

RUN	A	B	C	FSG		FGG		XG	
	$FeCl_3$ (mgL^{-1})	[GUM] (mgL^{-1})	ST (min)	Predicted RE(%)	Expt RE(%)	Predicted RE(%)	Expt RE(%)	Predicted RE(%)	Expt RE(%)
1	25	1	50	69.6	65.5±0.7	68.3	64.5±0.7	69.7	65.5±0.7
2	25	120	50	59.1	45.5±0.7	63.1	67±1.4	38.3	45.5±0.7
3	50	1	50	34.3	0±0.0	3.8	0±0.0	7.2	0±0.0
4	50	120	50	53.4	32±2.8	3.7	7.50±0.7	27.8	32±2.8
5	25	60.5	10	53.1	61±1.4	58.2	60±0.0	58.8	61±1.4
6	25	60.5	90	75.62	67.5±0.7	66.9	65±0.0	72.7	67.5±0.7
7	50	60.5	10	41.4	29.5±0.7	0.0	1±0.0	24.3	29.5±0.7
8	50	60.5	90	46.4	32±0.0	2.3	0.5±0.7	34.2	32±0.0
9	37.5	1	10	67.8	66±1.4	74.6	76.5±0.7	63.9	66±1.4
10	37.5	1	90	84.3	78.5±0.7	78.3	84±0.0	69.1	78.5±0.7
11	37.5	120	10	74.8	42.5±0.7	69.7	64±1.4	51.8	42.5±0.7
12	37.5	120	90	85.8	68.5±0.7	77.9	76±1.4	70.5	68.5±0.7
13	37.5	60.5	50	89.9	88.5±0.7	82.5	85.5±0.7	88.0	88.5±0.7
14	37.5	60.5	50	89.9	89±0.0	82.5	88.5±0.7	88.0	89±0.0
15	37.5	60.5	50	89.9	88±0.0	82.5	87±0.0	88.0	88±0.0

Table S8. Box-Behnken experimental design matrix of three variables along with the experimental and calculated removal efficiency (S2; Arsenate) at 295 K.

RUN	A	B	C	FSG		FGG		XG	
	FeCl3 (mg L^{-1})	[GUM] (mg L^{-1})	ST (min)	Predicted RE(%)	Expt RE(%)	Predicted RE(%)	Expt RE(%)	Predicted RE(%)	Expt RE(%)
1	25	1	50	36.9	42.5±0.7	39.1	39.5±0.7	57.8	58.5±0.7
2	25	120	50	50.7	49±0.0	36.3	34±0.0	25.1	22.50.7
3	50	1	50	42.3	44±0.0	9.2	11.5±0.7	31.4	34±1.4
4	50	120	50	28.1	22.5±0.7	24.9	24.5±0.7	14.7	14±1.4
5	25	60.5	10	40.1	41.5±0.7	39.0	40.5±0.7	49.1	51.5±0.7
6	25	60.5	90	56.3	51±0.0	41.6	42±0.0	54.0	53.5±0.7
7	50	60.5	10	35.7	41±1.4	17.4	17±0.0	31.0	31.5±0.7
8	50	60.5	90	43.4	42±1.4	21.9	20.5±0.7	35.4	33±0.0
9	37.5	1	10	29.1	22±0.0	9.4	7.5±0.7	55.6	52.5±0.7
10	37.5	1	90	52.3	52±0.0	14.3	13.5±0.7	54.7	54.5±0.7
11	37.5	120	10	40.2	40.5±0.7	17.2	18±1.4	25.3	25.5±0.7
12	37.5	120	90	40.9	48±1.4	19.6	21.5±0.7	35.4	38.5±2.1
13	37.5	60.5	50	76.2	76±0.0	67.5	67.5±0.7	66.7	67±2.8
14	37.5	60.5	50	76.2	77±0.0	67.5	67±0.0	66.7	66.5±0.7
15	37.5	60.5	50	76.2	75.5±0.7	67.5	68±0.0	66.7	66.5±2.1

Table S9. Observed and predicted organic arsenic removal efficiency for S1 (validation) at 295 K.

Test	GUM	Independent variable			As removal (%)		
		$FeCl_3$ dose (mgL^{-1})	GUM dose (mgL^{-1})	Settle time	Experimental	Predicted	% Error*
1	FSG	25	50.5	10	58.6±0.3	53.7	8.4
2	FSG	28.5	1	60	80.7±0.2	81.16	0.6
3	FSG	30	3	35	77.8±0.7	76.7	1.4
4	FGG	25	50.5	10	59.8±0.5	58.7	1.8
5	FGG	28.5	1	60	81.6±0.3	83.2	2.0
6	FGG	30	3	35	83.6±0.4	82.8	1.0
7	XG	25	50.5	10	59.4±0.8	60.5	1.9
8	XG	28.5	1	60	78.1±0.5	78.3	0.3
9	XG	30	3	35	77.6±3.3	77.0	0.8

*% Error for experimental vs predicted value with less than 5% error.

Table S10. Observed and predicted inorganic arsenic removal efficiency S2 (validation) at 295 K.

Test	GUM	Independent variable			As removal (%)		
		$FeCl_3$ dose (mgL^{-1})	GUM dose (mgL^{-1})	Settle time	Experimental	Predicted	% Error*
1	FSG	27	1	35	36.2±0.6	35.6	1.7
2	FSG	29.5	2.5	10	20.8±0.2	20.2	2.9
3	FSG	32	1.75	60	55.8±0.1	54.8	1.8
4	FGG	27	1	35	35.5±0.5	35.8	0.8
5	FGG	29.5	2.5	10	14.4±0.3	14.2	1.4
6	FGG	32	1.75	60	38.5±0.3	39.2	1.8
7	XG	27	1	35	59.8±1.1	60.5	1.2

8	XG	29.5	2.5	10	56.9±0.3	57.1	0.4
9	XG	32	1.75	60	63.1±0.7	63.7	1.0

*% Error for experimental vs predicted value with less than 5% error.
